# Approaches to multiplicity in publicly funded pragmatic randomised controlled trials: a survey of clinical trials units and a rapid review of published trials

**DOI:** 10.1186/s12874-022-01525-9

**Published:** 2022-02-06

**Authors:** Katie Pike, Barnaby C. Reeves, Chris A. Rogers

**Affiliations:** Bristol Trials Centre, Bristol Medical School, University of Bristol, Level 7, Zone A, Bristol Royal Infirmary, Bristol, UK

**Keywords:** Rapid review, Survey, Multiple testing, Randomised controlled trials

## Abstract

**Background:**

Opinions and practices vary around the issue of performing multiple statistical tests in randomised controlled trials (RCTs). We carried out a study to collate information about opinions and practices using a methodological rapid review and a survey, specifically of publicly funded pragmatic RCTs that are not seeking marketing authorisation. The aim was to identify the circumstances under which researchers would make a statistical adjustment for multiplicity.

**Methods:**

A review was performed extracting information from articles reporting primary analyses of pragmatic RCTs in one of seven high quality medical journals, in January to June (inclusive) 2018. A survey (Survey Monkey) eliciting opinions and practices around multiplicity was distributed to the 47 registered clinical trials units (CTUs) in the UK.

**Results:**

One hundred and thirty-eight RCTs were included in the review, and survey responses were received from 27/47 (57%) CTUs. Both the review and survey indicated that adjusting for multiplicity was considered most important for multiple treatment comparisons; adjustment was performed for 11/23 (48%) published trials, and 24/27 (89%) CTU statisticians reported they would consider adjustment. Opinions and practices varied around adjustment for multiplicity arising from multiple primary outcomes and interim analyses. Adjustment was considered less important for multiplicity due to multiple secondary outcomes (adjustment performed for 17/136 [13%] published trials and 3/27 [11%] CTU statisticians would consider adjustment) and subgroup analyses (8/85 [9%] published trials adjusted and 6/27 CTU [22%] statisticians would consider adjustment).

**Conclusions:**

There is variation in opinions about adjustment for multiplicity among both statisticians reporting RCTs and applied statisticians working in CTUs. Further guidance is needed on the circumstances in which adjustment should be considered in relation to primary trial hypotheses, and if there are any situations in which adjustment would be recommended in the context of secondary analyses.

**Supplementary Information:**

The online version contains supplementary material available at 10.1186/s12874-022-01525-9.

## Background

Multiple testing in randomised controlled trials (RCTs) occurs in situations including (but not restricted to): comparing multiple outcomes, performing multiple treatment comparisons (i.e. more than two treatment groups), analysing subgroups and interim analyses [1–3]. Multiplicity is a problem as it increases both the Type I (i.e. chance of finding false positive associations) and Type II (i.e. chance of false negative associations) error rates in significance testing; which could result in either recommending a treatment that doesn’t work, or missing an important treatment effect [1]. The consequences of this may be different for publicly funded pragmatic RCTs seeking to address important uncertainties experienced by healthcare professionals, than for commercially-funded trials carried out for drug-licensing purposes.

Trials that define multiple primary outcomes can be designed to require either: a) all primary outcomes to meet pre-defined effectiveness criteria for the new treatment to be declared effective, or b) at least one primary outcome to demonstrate effectiveness for treatment to be declared effective [4, 5]. In addition, most trials also analyse data for a number of secondary outcomes, requiring multiplicity to be considered [1, 4].

Multiple treatment comparisons arise when participants are randomised to one of more than two groups, meaning multiple pairwise comparisons are made. Treatments in such a trial may be related (e.g. a dose-response trial) or distinct (e.g. comparing two unrelated treatments to a common control group). Questions remain, for example should practice vary in different scenarios? [6].

A subgroup analysis involves testing for a treatment by subgroup interaction [7]. Such analyses are typically carried out for multiple subgroups. Hypotheses about the interactions may be, but are not always, prespecified. Subgroup analyses, whether one or many, create multiplicity because they are in addition to the primary comparison.

Finally, interim analyses relate to repeatedly testing for the same treatment effect at multiple interim reviews. Undertaking interim analyses inflates the overall trial false-positive rate, the magnitude of this inflation depends on the number of interim analyses performed [3].

Proposed solutions consist of some form of statistical adjustment for multiple tests; either by adjusting the significance level for inference such that the probability of a false-positive finding reduces appropriately, or performing tests in a hierarchical manner [8, 9]. Opponents of such adjustments state that, in general, they are unhelpful and lack substance [2]; a given comparison will be interpreted differently according to the number of other tests performed, and that the compromise of reducing the Type I error is to increase Type II error [10]. Both the European Medicines Agency (EMA) and US based Food and Drugs Administration (FDA) have published guidance on when adjustment should be implemented, which is mandatory for RCTs carried out to support an application for marketing authorisation [11, 12]. However, there is no comparable guidance for publicly funded trials that are not seeking marketing approval.

### Objectives

We aimed to describe existing practices and approaches to address multiplicity in publicly funded, pragmatic RCTs, under various scenarios that create multiplicity issues. We focussed on eliciting both the approaches taken in RCTs recently published in high-quality medical journals and the opinions of applied statisticians and methodologists working on the design and analysis of such trials. To achieve this aim, we performed:

1. A rapid review of RCTs recently published in seven high-impact medical journals
2. A survey of statisticians working in UK Clinical Research Collaboration (UKCRC) registered clinical trials units (CTUs)

## Methods

### Rapid review

#### Eligibility criteria

Articles were included if they met the following inclusion criteria: RCT of any design (defined as participants/clusters of participants allocated at random to receive an intervention); primary analysis of an RCT (excluding secondary analyses/reports, and longer term follow-up of RCT participants); effectiveness RCTs (excluding pilot, feasibility, exploratory, phase I, phase II trials, or trials with fewer than 100 participants); publicly funded sponsored/funded RCTs; published in January to June (inclusive) 2018 in either Annals of Internal Medicine, British Medical Journal (BMJ), Journal of the American Medical Association (JAMA), Lancet, New England Journal of Medicine (NEJM), National Health Technology Assessment (HTA) or PLOS Medicine (PLoS Med). The eligibility criteria were designed to capture RCTs meeting the aims of the study. The choice to restrict the review to seven general medical journals with high impact factors was to ensure that, in the authors’ opinion, publicly funded, pragmatic RCTs were included that were highly likely to have undergone independent, rigorous, statistical review. As a result, there should have been less use of inappropriate methods (which would dilute the findings of the review). This approach has been used in a previous review [6].

#### Information sources and search

Potentially eligible studies were identified via a Medline search (performed on 30/07/2019):

1. (annals of internal medicine).jn or (bmj).jn or (jama).jn or (lancet).jn or (new england journal of medicine).jn or (health technology assessment winchester England).jn or (plos medicine public library of science).jn
2. Limit 1 to (adaptive clinical trial or clinical trial, all or clinical trial, phase i or clinical trial, phase ii or clinical trial, phase iii or clinical trial, phase iv or clinical trial or controlled clinical trial or randomized controlled trial)^1^
3. (annals of internal medicine).jn^2^
4. (trial or prospective study).ti
5. 3 and 4
6. 2 or 5
7. limit 6 to (english language)
8. limit 7 to yr = “2018”

#### Study selection

Eligibility screening was performed within EndNote. Abstracts were screened to exclude articles published between July and December 2018, and then full-text articles were screened to exclude based on the other characteristics (secondary report/analysis of RCT, editorial/commentary article on an RCT, non-RCT design, early phase RCTs, commercially sponsored RCT).

#### Data collection process

An Access database was developed, piloted on 20 randomly selected articles and then refined accordingly. See Additional file 1 for the data collection form.

#### Data items

The number of primary/secondary outcomes stated in the article’s methods section and the number of primary/secondary comparisons performed in the article’s results section were recorded. Occasionally the number of primary outcomes stated differed from the number of comparisons performed, due to e.g. comparisons made at multiple time point or adjusted and unadjusted analyses performed, and it being unclear which was the primary comparison. We also recorded whether the trial was considered (by the reviewers) to require either: a) all primary outcomes or b) at least one primary outcome to meet effectiveness criteria for the treatment to be declared effective. For example in the case of two endpoints A and B, whether a) both A and B, or b) either A or B must reach the pre-specified criteria for effectiveness for the treatment to be declared effective. This distinction was made as adjustments for multiplicity are often considered unnecessary in the case of effectiveness being required for all primary outcomes [4].

The number of treatment group comparisons made and whether the treatments were defined (by the reviewers) as related or distinct were recorded. Treatments were classified as related if, for example, they were different doses or different treatment schedules of the same (or related) interventions. For example, comparing: a) weekly administration of a therapy with control treatment, and b) monthly administration of a therapy with control treatment. For treatments to be classified as distinct the interventions must have been entirely different to each other (although they may have been compared to the same control group). For example, comparing: a) a behavioural therapy with control treatment, and b) a drug therapy with control treatment. This distinction was made as some methodologists believe multiplicity adjustments may be less necessary in the case of distinct treatment groups [1, 6].

The number of subgroup analyses (and whether they were pre-specified or post-hoc) and the number of interim analyses were also recorded. Finally, any other analyses that led to multiplicity (e.g. sensitivity, post-hoc, ancillary or exploratory analyses) were recorded. For each of the above areas details of any adjustment method(s) implemented for multiple testing was recorded, along with any justification given.

#### Summary measures and synthesis of results

Findings were summarised in descriptive tables, summarising the approach taken to multiplicity for each area. Data management and analysis was performed in Stata (version 15).

### Survey

The survey was drafted, piloted on a small number of CTUs and individuals, and revised following feedback. A copy of the survey is included in Additional file 2. Topics covered included: existing practices to address multiplicity; the approaches CTUs would take to different specified scenarios (covering multiple outcomes, multiple treatment comparisons, subgroup analyses, interim analyses and other design considerations); adjustment methods used; the proportion of trials carried out with certain attributes that introduce multiplicity (e.g. multiple outcomes, multiple treatment comparisons, etc); and thoughts on the primary problem areas regarding multiplicity.

The survey was implemented via Survey Monkey and distributed to the 47 CTUs with full registration status in the UKCRC Registered CTU Network [13]. The UKCRC CTU statistics group kindly distributed the survey to the nominated statistician from each unit. The survey findings were then collated and analysed using Stata (version 15).

## Results

### Rapid review

#### Study selection

**Fig. 1:**
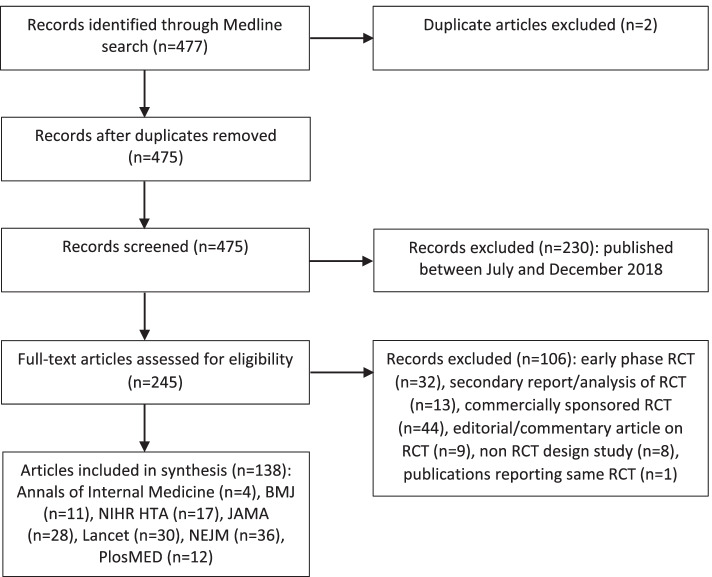
Review: screening process

A total of 247 articles were identified and assessed for eligibility; 108 articles were excluded during the screening process, therefore 139 articles reporting on 138 RCTs were included (Fig. 1).

#### Study characteristics

Table 1 summarises the study characteristics. Approx. two-thirds (68%) of the trials used a parallel group design with two treatment groups, very low numbers of trials used crossover or factorial designs. Most trials were individually randomised; 17% were cluster randomised and two were stepped wedge trials. Most trials tested superiority hypotheses; 13% tested non-inferiority hypotheses and one trial tested equivalence. The median number of randomised patients in each trial was 574 (interquartile range [IQR] 312–2043). In terms of multiplicity concerns, 21/138 trials (15%) stated more than one primary outcome in the study methods and 28/138 (20%) made more than one primary outcome comparison. There were 136/138 (99%) studies with either at least two secondary outcomes stated or at least two secondary outcome comparisons made. In total 23/138 (17%) of trials had three or more treatment groups. The majority of trials (85/138; 62%) described subgroup analyses, and 38/135 (28%) trials had performed interim analyses. The characteristics of trials with/without each source of multiplicity are compared in Additional file 3, Tables S1 to S4.

**Table 1:** Review: study characteristics

Characteristic		n/N	%
Journal	Annals of Internal Medicine	4/138	3%
	BMJ	11/138	8%
	JAMA	28/138	20%
	Lancet	30/138	22%
	NEJM	36/138	26%
	NIHR HTA journal library	17/138	12%
	PlosMED	12/138	9%
Trial design^a^	Parallel group: 2 treatment groups	94/138	68%
	Parallel group: > 2 treatment groups	18/138	13%
	Cluster randomised	23/138	17%
	Crossover	2/138	1%
	Factorial	5/138	4%
	Stepped wedge	2/138	1%
	Non-inferiority	18/138	13%
	Equivalence	1/138	1%
Total number of randomised participants - median (IQR)		574	(312, 2043)
**MULTIPLICITY**			
Primary outcome^b^	More than one outcome stated	21/138	15%
	Two outcomes	17	
	Three outcomes	4	
	More than one comparison made	28/138	20%
	Two comparisons	16	
	Three comparisons	4	
	Four comparisons	2	
	Five comparisons	1	
	>Five comparisons (maximum 20)	5	
Secondary outcome	More than one outcome stated	134/138	97%
	Median (IQR) outcomes stated	8 (5, 13)	
	More than one comparison made	132/138	96%
	Median (IQR) comparisons made	14.5 (7, 26)	
More than two treatment groups^c^		23/138	17%
Number of treatment comparisons made	One^d^	1	
	Two	13	
	Three	6	
	Four	1	
	Five	1	
	Eight	1	
Any subgroup analyses performed		85/138	62%
	Median (IQR) subgroup analyses	4 (2, 7)	
Any interim analyses performed		38/135	28%
	One	22	
	Two	9	
	Three	3	
	Four	3	
	Five	1	

*Notes*: ^a^ Trials could be classified in more than one design category, e.g. a cluster randomised, factorial, non-inferiority trial.

^b^Discrepancies between the numbers of outcomes stated and comparisons made were either due to multiple time points being analysed or multiple analysis approaches taken, with none stated as primary.

^c^This includes parallel group trials with > 2 treatment groups and factorial trials.

^d^In this trial one treatment arm was dropped due to futility at an interim analysis, so the final analysis comprised just two treatment groups and therefore one comparison.

*Abbreviations*: *BMJ* British Medical Journal, *IQR* interquartile range, *JAMA* Journal of the American Medical Association, *NEJM* New England Journal of Medicine, *NIHR HTA* National Institute of Health Research Health Technology Assessment, *PlosMED* Public Library of Science Medicine

#### Synthesis of results

#### Multiple outcomes

In total 28 studies had multiple primary outcomes. For eight of these RCTs effectiveness was required for all primary outcomes, of which adjustment was performed for two trials (hierarchical testing methods). For the remaining 20 studies effectiveness was required for at least one primary outcome; seven of these RCTs adjusted for multiplicity (either a formal procedure (Bonferroni or Holm) [8]; hierarchical testing methods; *p*-values between 0.025 and 0.05 considered to have borderline significance; or performing a post-hoc analysis with 97.5% confidence intervals). In terms of secondary outcomes, adjustment for multiplicity was performed for 17 (12%) of the trials reviewed: either a formal procedure (Bonferrroni, Holm, Hochberg, using a 1% threshold for significance or a graphical method) [8]; hierarchical testing or restricting formal hypothesis testing to just key secondary outcomes. See Table 2a for full details.

**Table 2:** Approach to multiplicity due to multiple outcomes

**a) Review: multiplicity approach taken**				
	**Formal adjustment**	**Hierarchical testing**	**Other approach**	**None**
**PRIMARY OUTCOMES**				
All outcomes to be declared effective	0/8	2/8^a^	0/8	6/8
One or more outcomes to be declared effective^b^	4/20^c^	1/20^d^	2/20^e^	13/20
**SECONDARY OUTCOMES**				
Secondary outcomes	14/136^f^ (10%)	1/136^g^ (1%)	2/136^h^ (1%)	119/136 (88%)
**b) Survey: responses to posed scenarios**				
		**Yes**	**No**	**Unsure**
**PRIMARY OUTCOMES**				
Consider a parallel group trial with two primary outcomes. Would you adjust for multiplicity in the following scenarios?				
The trial hypotheses require both null hypotheses to be rejected?		9/27 (33%)	16/27 (59%)	2/27 (7%)
The trial hypotheses require either null hypothesis to be rejected?		16/27 (59%)	8/27 (30%)	3/27 (11%)
**SECONDARY OUTCOMES**				
Would you adjust for multiplicity arising from multiple secondary outcomes?		3/27 (11%)	20/27 (74%)	4/27 (15%)
Would the type of outcomes (efficacy, safety, cost-effectiveness) have an impact on your response to the above question?		9/27 (33%)	17/27 (63%)	1/27 (4%)

*Notes:* ^a^Both trials sequentially tested two outcomes

^b^ Includes 13 trials that stated multiple primary outcomes in the methods section, and seven that stated only one outcome but made multiple comparisons

^c^ Two trials performed a Holm correction, and two implemented a graphical multiple testing procedure

^d^ Trial sequentially tested non-inferiority then superiority

^e^ One trial recommended that p-values between 0.025 and 0.05 were considered to have borderline significance; one trial performed post-hoc analysis of the primary outcomes with one-sided 97.5% confidence intervals

^f^ Three trials performed a Bonferroni correction, five a Holm correction, one a Hochberg correction, three used a 1% threshold for significance and two used a graphical method

^g^ Formal hypothesis testing was only performed for secondary outcomes if the primary efficacy outcome was statistically significant

^h^ Formal hypothesis testing was only performed for a small number of key secondary outcomes, other secondary outcomes were just presented descriptively

#### Multiple treatment comparisons

Twenty-three trials made multiple treatment comparisons. Fifteen of these were classified as having related treatments of which nine adjusted for multiplicity (either using a formal procedure (Bonferroni, Holm, Hochberg or using a 1% significance level for all treatment comparisons) [8]; performing hierarchical analysis; or making allowance for multiplicity in the interpretation by performing a post-hoc Bonferroni correction). Of the eight trials that compared distinct treatments, two adjusted for multiplicity (Bonferroni or Dunnett’s procedures) [8]. See Table 3a.

**Table 3:** Approach to multiplicity due to multiple treatment comparisons

**a) Review: multiplicity approach taken**				
	**Formal adjustment**	**Hierarchical testing**	**Other approach**	**None**
Related treatments	7/15^a^	1/15^b^	1/15^c^	6/15
Distinct treatments	2/8^d^	0/8	0/8	6/8
**b) Survey: responses to posed scenarios**				
		**Yes**	**No**	**Unsure**
Would you consider adjusting for multiplicity arising from making multiple treatment comparisons?		24/27 (89%)	1/27 (4%)	2/27 (7%)
Consider a parallel group trial with three treatment arms, where all comparisons are of interest. Would you adjust for multiplicity in the following scenarios?				
Two of the treatment arms are related, e.g. Group 1 = placebo, Group 2 = low drug dose, Group 3 = high drug dose		22/27 (81%)	1/27 (4%)	4/27 (15%)
The three treatment arms are unrelated, including one placebo arm, e.g. Group 1 = placebo, Group 2 = drug, Group 3 = exercise		16/27 (59%)	7/27 (26%)	4/27 (15%)
The three treatment arms are unrelated, but all are active treatments, e.g. Group 1 = drug, Group 2 = exercise, Group 3 = education		19/27 (70%)	6/27 (22%)	2/27 (7%)
Would you be more likely to adjust for multiplicity if the number of treatment arms was increased?		12/27 (44%)	12/27 (44%)	3/27 (11%)

*Notes*: ^a^ Three trials performed a Bonferroni correction, one a Holm correction, two a Hochberg correction and one used a 1% significance level for all treatment comparisons

^b^ Two treatment comparisons were split into primary and secondary hypotheses and analysed in a hierarchical manner

^c^ A post-hoc Bonferroni correction was performed, although this was not the primary analysis for the trial

^d^ One trial performed a Bonferroni correction and one used Dunnett’s procedure

#### Subgroup analyses

Most studies (77/85; 91%) did not make any allowance for multiplicity from subgroup analyses. The eight trials that adjusted for multiplicity used either formal procedures (Bonferroni or Holm) [8] or a 1% threshold for subgroup analyses. See Table 4a.

**Table 4:** Approach to multiplicity due to subgroup analyses

**a) Review: multiplicity approach taken**				
	**Formal adjustment**	**Hierarchical testing**	**Other approach**	**None**
Subgroup analyses	8/85^a^ (9%)	0/85 (0%)	0/85 (0%)	77/85^b^ (91%)
**b) Survey: responses to posed scenarios**				
		**Yes**	**No**	**Unsure**
Would you consider adjusting for multiplicity arising from performing multiple subgroup analyses?		6/27 (22%)	17/27 (63%)	4/27 (15%)
Consider a parallel group trial with multiple subgroup analyses performed. Would you adjust for multiplicity in the following scenarios?				
Subgroup analyses pre-specified in the study protocol?		3/27 (11%)	22/27 (81%)	2/27 (7%)
Subgroup analyses determined post-hoc?		4/27 (15%)	22/27 (81%)	1/27 (4%)
Subgroup analyses specified for the following reasons: a) to confirm biological plausibility, b) to confirm existing hypotheses, AND c) to show subgroup effects for supporting decision making in target populations.		3/27 (11%)	19/27 (70%)	5/27 (19%)
Would you be more likely to adjust for multiplicity if the number of subgroup analyses was increased?		5/27 (19%)	21/27 (78%)	1/27 (4%)

*Notes:* ^a^ One trial performed a Bonferroni correction, two a Holm correction and five studies used a threshold of 1% for significance

^b^ Of these, five studies stated that results from secondary outcomes were exploratory/hypothesis generating

#### Interim analyses

Of the 41 trials that had performed interim analyses, approximately two-thirds (28/41; 68%) made some allowance for multiplicity. Most of these trials used a formal procedure. See Table 5a.

**Table 5:** Approach to multiplicity due to interim analyses

**a) Review: approach taken to multiplicity**				
	**Formal adjustment**	**Hierarchical testing**	**Other approach**	**None**
Interim analyses	26/41^a^ (63%)	0/41 (0%)	2/41^b^ (5%)	13/41^c^ (32%)
**b) Survey: responses to posed scenarios**				
	**Always**	**Sometimes**	**Never**	**Unsure**
Would you adjust for multiplicity if interim analysis(es) were pre-specified in the study protocol?	8/27 (30%)	12/27 (44%)	3/27 (11%)	4/27 (15%)

*Notes*: ^a^ Eight trials used the Haybittle-Peto procedure, eight used O’Brien-Fleming, seven partitioned the significance level between final and interim analyses (with no further details given), one used Pocock, one used Lan DeMets and one did not give details.

^b^One trial used a group sequential design and one used a conditional rejection probability approach.

^c^Of these, three trials stated a pre-specified significance level for stopping the trial.

#### Other factors

Most studies (123/138, 89%) reported at least one further analysis that could be subject to multiplicity considerations (e.g. sensitivity, post-hoc, ancillary or exploratory analyses). Of these, five made some allowance for multiplicity. Very few trials gave any justification for their approach to multiplicity, other than providing a generic caution in the Methods of the reports, e.g. “*The secondary end points and the sensitivity analyses were not adjusted for multiple testing; therefore, the results of these analyses should be considered exploratory*” [14].

### Survey

Survey responses were received from 27/47 (57%) CTUs. See Additional file 3, Table S5 for a summary of the existing practices in CTUs to address multiplicity. Most CTUs determine the approach to multiplicity at the design stage (85%, 23/27), and take a bespoke approach to managing multiplicity (78%, 21/27). Nineteen (70%) said that their approach to multiplicity would (or would possibly) vary depending on how pragmatic the trial objectives are intended to be.

#### Multiple outcomes

In a posed trial with two primary outcomes a third (9/27) of CTUs would consider adjusting for multiplicity in the scenario whereby the trial hypotheses required both null hypotheses to be rejected, and just over half (16/27) in the alternative scenario whereby rejection of either null hypothesis was sufficient (Table 2b). Relatively few CTUs would adjust for multiplicity arising from secondary outcomes (3/27). Example comments were: “*As long as there is a ‘primary’ outcome, I don’t make any multiplicity adjustments for any secondary outcomes regardless of type of outcome*” and “*Multiple testing intrinsically suggests that you have power to detect clinically relevant differences in secondary outcomes - this isn’t always the case*”.

#### Multiple treatment comparisons

In terms of multiplicity arising from making multiple treatment comparisons, the majority of CTUs (24/27) would consider adjustment (Table 3b). Of the different scenarios posed, the nature of the comparisons had some bearing on how likely researchers were to adjust: 22/27 would consider it when treatment groups are related, 16/27 when groups are unrelated with one comprising a placebo group, and 19/27 for unrelated active groups. For 44% (12/27) of researchers more treatment groups would make them more likely to adjust for multiplicity. Comments arose around the nature of comparisons, for example: “*if only comparing each treatment arm against placebo, and interventions are unrelated, less likely to employ a correction*” and “*if treatments are unrelated, each paired comparison can be treated as a separate clinical trial even if they exist within the same trial*”. However, other comments arose around the use of a common control group “*the challenge is with the same common control group used for two pairwise comparisons*”.

#### Subgroup analyses

Most CTUs (17/27, and a further four were unsure) would not adjust for multiplicity arising from subgroup analyses, see Table 4b. Furthermore, the type of subgroup analysis (pre-specified, post-hoc etc) appeared to have little bearing on whether adjustment would be performed. Themes commonly emerging in the comments were around the exploratory nature and low power of such analyses, for example: “*Pre-specified subgroup analyses are usually undertaken to confirm any treatment effect across the groups and should be undertaken using interaction tests. These are not usually powered.”*.

#### Interim analyses

There was no consensus across CTUs regarding the use of adjustment for multiplicity arising from performing interim analyses (Table 5b). Example comments around the factors that influence decision making include: “*Exactly what analyses are being performed, whether it involves hypothesis testing, and whether it involves the primary outcome*”.

#### Other

None of the following factors appeared to have a strong influence on the decision making around adjustment for multiplicity: overall design (e.g. cluster, factorial, crossover); research question (e.g. superiority, non-inferiority, equivalence); intervention type (e.g. complex, behavioural, pharmacological); or an imbalanced trial allocation (Additional file 3, Table S6). An open-ended question *“What do you think are the common problem areas for multiplicity? Where is research needed?”* gave responses summarised in Table 6. The most common category of responses was “consensus/awareness amongst trialists/clinicians”. In terms of the methods CTUs used (Additional file 3, Table S7), the most common were Bonferroni and Dunnett [8]. Rarely used methods were Hommel, fallback and parallel gatekeeping [8].

**Table 6:** Survey: comments

Category	Example comments
**Consensus/awareness amongst trialists/clinicians (*n*** **= 11):** ranging from needing consensus on which methods to use when, understanding when multiplicity adjustments are required, and clinician awareness	*“Lack of consensus among statisticians leaves a lot of uncertainty and makes CIs uncomfortable”*
	*“Many trialists don’t know the different methods that can be used (or haven’t got the time to investigate their correct implementation) so a state of the art type review and a course for the most useful/suitable methods would be great”*
	*“Deciding when it is required and providing justification when the decision is not to adjust”*
**Informal hypothesis testing (*n*** **= 2):** including repeated presentation of primary outcome data by arm to DMCs, and data dredging	
**Confidence intervals (n = 2)**	*“We are supposed to be concentrating on measures of effect and confidence intervals, and downplaying p-values. How does this factor into multiplicity testing procedures?*”
**Multi-arm trials (n = 2):** including multiple treatment arms and adaptive trials	
**Multiple outcomes (n = 2):** including classifying the purpose of secondary outcomes, and risk/benefit synthesis	
**Subgroup analyses (n = 1)**	*“Design for subgroup effects on basis of meta-analysis including previous results”*
**Interim analyses (n = 1)**	*“Minimise interim analyses”*
**Miscellaneous (n = 2):** including the increased importance of personalised medicine and the lack of consensus of what the denominator is for significance	

*Abbreviations: CI* Chief Investigator, *DMC* Data Monitoring Committee

## Discussion

There are conflicting opinions and practices around whether an adjustment for multiple testing should be made in different scenarios in publicly funded pragmatic RCTs not seeking marketing authorisation. In both the review and survey there is a difference between multiplicity scenarios relating to primary study hypotheses (e.g. multiple primary outcomes and multiple treatment comparisons) and secondary hypotheses (e.g. secondary outcomes and subgroup analyses). In the case of the former, opinion is divided on adjustment, and decision making around whether to adjust depends on the context. For the latter, the consensus appears to be not to adjust for multiplicity.

When considering multiple primary outcomes, the methodological literature suggests adjustment for multiplicity is generally not needed when effectiveness is needed for all primary outcomes for the treatment to be declared effective, but should be considered when effectiveness is required only for one or more outcome [4, 5]. This was only partially reflected in the findings of this study. When all outcomes were required to meet effectiveness criteria, 2/8 studies in the review made an adjustment and 9/27 surveyed CTUs would consider adjustment. In the case of effectiveness being required for one or more outcomes 7/20 reviewed studies made an adjustment for multiplicity and 16/27 surveyed CTUs said they would consider it. Regulatory guidance from both the EMA and FDA for trials seeking marketing authorisation states that no adjustment to significance levels is required if effectiveness is needed for all primary outcomes, but adjustment is required if there is more than one way for a trial to “win” (i.e. any primary outcome can be declared effective) [11, 12].

In an analogous manner, in the context of multiple treatment comparisons the methodological literature suggests greater need for adjustment when comparing related treatments than distinct treatments [1, 6]. Again this was partially reflected in our study; in the review 9/15 studies adjusted for multiplicity due to related treatments and 2/8 for distinct treatments. From the survey only marginally more CTUs would consider adjustment for related treatments (22/27) than distinct treatments (16 or 19 CTUs depending on the nature of the comparison). Regulatory guidance in this area is less clear; the EMA guidance covers two specific examples (comparison of investigational drug, existing ‘reference’ drug and placebo; and dose-response trials) both of which would require adjustment [11]. The FDA guidance does not explicitly cover multi-group trials [6, 12].

The review demonstrated that few authors adjusted for multiplicity arising from secondary outcomes or subgroup analyses. This finding was mirrored in the survey, in which relatively few CTUs reported considering adjustment in these circumstances. This is likely to be because these analyses are usually viewed as exploratory, i.e. study conclusions are not based on them. This approach is endorsed by the methodological literature, which generally advocates such analyses being supportive in nature, and some publications suggest that the focus should be around whether findings are consistent with the overall findings of the trial, rather than significance testing per se [1, 4, 15]. Regulatory guidance suggests distinctions should be made between analyses intended to be supportive of the trial’s overall aims, and those that are intended to lead to a claim of effectiveness in their own right [11, 12, 16]. Indeed, one high impact medical journal states that if adjustment is not implemented to control Type I error in such analyses, reporting should be limited to point estimates and associated 95% confidence intervals, i.e. *p*-values should not be given [17]. However, in the context of publicly funded pragmatic trials the triallists aren’t necessarily controlling how the conclusions are interpreted and how the evidence is used. Guidance groups and clinicians may take a wider view about evidence than just the primary outcome. This leads to questions around whether there are scenarios when adjustment for multiplicity may be sensible for secondary outcomes or analyses.

Practice around multiplicity adjustments in publicly funded pragmatic RCTs differs from the recommendations made in regulatory guidance. In our opinion this reflects the contrast between trials done to obtain marketing authorisation and trials done to address effectiveness claims. For trials seeking marketing authorisation the need for a clear dichotomous answer (approval/no approval) aligns with the hypothesis testing paradigm (accept/reject), and therefore these trials drive much of the activity in multiplicity adjustments [2]. Pragmatic trials may be designed to differentiate between a number of important uncertainties experienced by health care professionals, as well as considering a cost-effectiveness analysis. In the decision making around multiplicity adjustments, we should consider the financial implications of recruiting more patients to allow for reduced Type I and/or Type II error rates. A systematic review in 2016 estimated the average per patient cost to be $409 (with a large range of $41 to $6990) [18].

Limitations of the review are that due to its focus on all pragmatic RCTs, relatively few studies with multiple primary outcomes or performing multiple treatment comparisons were included. A future extension would be a review with narrower inclusion criteria focusing specifically on studies with multiple primary outcomes or multiple treatment groups. Restricting the review to seven major journals is a potential limitation due to the potential impact on representativeness. A limitation of the survey is the moderate response rate; it would be useful to have the views of more CTUs, and perhaps an international perspective. Furthermore, in a future survey it may be useful to have more detailed questions regarding practices around informal hypothesis testing.

## Conclusions

There is variation in both the approach that has been taken to address multiplicity in publicly funded, pragmatic RCTs published in high-quality medical journals and the opinions of applied statisticians working in CTUs. In the situation where multiplicity arises as part of the primary hypotheses of the trial, adjustment is more likely (albeit not at all guaranteed) to be performed than when multiplicity arises amongst secondary hypotheses. Further work and guidance is needed around: a) the specific circumstances in which adjustment should be performed in relation to primary trial hypotheses, b) if there are any situations in which adjustment would be recommended in the context of secondary analyses.

## Supplementary Information

**Additional file 1.** Access database used for data collection in the review.

**Additional file 2.** Copy of CTU survey.

**Additional file 3: Table S1.** Review: characteristics of trials that did/did not have multiple primary outcomes. **Table S2.** Review: characteristics of trials that did/did not perform multiple treatment comparisons. **Table S3.** Review: characteristics of trials that did/did not perform subgroup analyses. **Table S4.** Review: characteristics of trials that did/did not perform interim analyses. **Table S5.** Survey: existing practices in CTUs to address multiplicity. **Table S6.** Survey: effect of other trial design features on the approach to multiplicity. **Table S7.** Survey: statistical methods used to address multiplicity.

## Data Availability

The datasets used and/or analysed during the current study are available from the corresponding author on reasonable request.

## Abbreviations

BMJ: British Medical Journal
CTU: Clinical Trials Unit
EMA: European Medicines Agency
FDA: Food and Drugs Administration
HTA: Health Technology Assessment
IQR: Interquartile Range
JAMA: Journal of the American Medicine Association
NEJM: New England Journal of Medicine
PLoS Med: PLOS Medicine
RCT: Randomised Controlled Trial
UKCRC: UK Clinical Research Collaboration

## Footnotes

1 Although early phase trials were excluded from the review, the search strategy included trials indexed as phase I/II to ensure that any effectiveness trials erroneously indexed as phase I/II were captured. Early phase trials according to the review’s exclusion criteria were then excluded at the full-text screening stage.

2 This journal was listed separately as many Annals of Internal Medicine articles did not have relevant clinical trial publication types (i.e. those given in point 2) indexed in Medline
