# Supplementary material for: Approaches to multiplicity in publicly funded pragmatic randomised controlled trials: a survey of clinical trials units and a rapid review of published trials
Source: BMC Med Res Methodol. 2022 Feb 6;22:39. doi: 10.1186/s12874-022-01525-9 (PMC8818238; doi:10.1186/s12874-022-01525-9)
Supplement: Supplementary file 1 — Additional file 1. Access database used for data collection in the review. [file 12874_2022_1525_MOESM1_ESM.pdf]

Additional file 1

Access database used for data collection in the review

Page 1: General information

GENERAL INFORMATION

Manuscript reference number	(New)
Clinical trials registration number	
Journal	
Lead author	
Title	
Citation	
Population	
Trial design	
Other trial design info	
Allocation ratio	
Trial phase	
Total number of randomised participants	0
Interventions	
Trial conclusions	

Page 2: Multiplicity due to outcomes

PRIMARY OUTCOME(S)

Primary outcome(s)	
Number of primary outcomes stated	0
Number of primary outcome comparisons made	0
Discrepancies in numbers of outcomes/comparisons	
Table numbers (primary)	
Statistical approach to multiplicity (primary outcomes)	
Further multiplicity details, primary	
Number of other comparisons made related to primary outcc	0
Details of such comparisons	
Any other info, primary	
Co-primary outcomes	

SECONDARY OUTCOMES

Number of secondary outcomes stated	0
Number of secondary outcome comparisons made	0
Discrepancies in numbers of secondary outcomes/comparisons	
Tables numbers (secondary)	
Statistical approach to multiplicity (secondary outcome)	
Further multiplicity details, secondary	
Any other info, secondary	

REPEATED MEASURES

Repeated measurements for any outcomes	
Already counted in primary/secondary comparisons?	☐
Statistical approach to multiplicity (repeated measurements)	
Further multiplicity details, repeated	
Any other info, repeated	

Page 3: Multiplicity due to other factors

TREATMENT GROUPS

Number of treatment groups	0
Statistical approach to multiplicity (multiple treatments)	
Further multiplicity details, treatments	
Any other info, treatments	
Number of comparisons	0
Description of comparisons	
Comparisons related?	☐

SUBGROUP ANALYSES

Number of pre-specified subgroup analyses reported	0
Number of post-hoc subgroup analyses reported	0
Outcomes subgroup analyses performed for	
Statistical approach to multiplicity (subgroups)	
Further multiplicity details, subgroups	
Any other info, subgroups	

INTERIM ANALYSES

Interim analyses mentioned?	☐
Number of interim analyses performed	0
Statistical approach to multiplicity (interim)	
Further multiplicity details, interim	
Any other info, interim	

OTHER

Other multiplicity concerns	
Statistical approach to multiplicity (other)	
Further multiplicity details, other	
Any justification for multiplicity approach	
Any other relevant information	
