# Supplementary material for: Approaches to multiplicity in publicly funded pragmatic randomised controlled trials: a survey of clinical trials units and a rapid review of published trials
Source: BMC Med Res Methodol. 2022 Feb 6;22:39. doi: 10.1186/s12874-022-01525-9 (PMC8818238; doi:10.1186/s12874-022-01525-9)
Supplement: Supplementary file 2 — Additional file 2. Copy of CTU survey. [file 12874_2022_1525_MOESM2_ESM.pdf]

Additional file 2

Copy of CTU survey

INTRODUCTION

The objective of this survey is to understand existing practices in CTUs for addressing multiplicity in RCTs. In particular, I am interested in exploring whether approaches used differ according to the “cause” of the multiplicity (e.g. testing multiple outcomes, testing in multiple subgroups, etc).

Please read the following points before completing the survey:

1. The approach taken to address multiplicity issues in RCTs can be considered to fall into two categories:

- Implementing (or not) some form of **statistical multiple testing procedure** (e.g. adjustment of p-values, testing hierarchically ordered hypotheses)
- Taking consideration of multiplicity issues within the **interpretation** of RCT findings

The focus of this survey is on the use of multiple testing procedures rather than the interpretation of results.

2. Some questions refer to pragmatic RCTs only. For the purposes of this survey a **pragmatic RCT** is defined as a “larger” RCT designed to test the effectiveness of the intervention in broad routine clinical practice. I.e. the results are expected to be generalisable to the wider NHS.

3. The answers to some of the questions may vary according to trial, therapeutic area and possibly other factors (e.g. opinions of the other trialists or clinicians). Where possible, please give the approach used within your CTU. If that is not possible, please give your opinion of best-practice.

* Name of CTU (please select):

Role/job title of person completing the survey:

EXISTING PRACTICES TO ADDRESS MULTIPLICITY

This section focuses on existing procedures and policies to address multiplicitywithin your CTU.

At what stage is the approach to address multiplicity in RCTs usually determined in your CTU?

○ Design (stated in grant application) ○ Design (stated in protocol) ○ Design (stated in SAP) ○ Analysis ○ Varies ○ Never determined

If determined at the design stage, have there been circumstances when post-hoc decisions have been made about multiplicity?

○ Yes ○ No ○ Unsure

If yes, please give further details:

Is the approach to multiplicity in your CTU?

○ Standard across all trials ○ Bespoke approach that varies from trial to trial ○ Other

Please give further details (if possible):

Would your approach to multiplicity vary according to how pragmatic the trial is (e.g. early phase efficacy vs later phase effectiveness trials)?

○ Yes ○ Possibly ○ No ○ Unsure

Please give further details (if possible):

Multiple testing in RCTs

APPROACHES TO ADDRESS MULTIPLICITY: MULTIPLE OUTCOMES

This section focuses on pragmatic RCTs (i.e. "larger" trials where there is an expectation that results will be generalisable to the wider NHS).

Would you consider implementing a multiple testing procedure to address multiplicity arising from measuring multiple outcomes (primary and/or secondary)?

◯ Yes ◯ No ◯ Unsure

If **YES**, complete the rest of this section. If **NO**, go to next section.

Consider a parallel group trial (two treatment arms) with two primary outcomes. Would you implement a multiple testing procedure for the primary outcomes in the following scenarios?

	Yes	No	Unsure
The trial hypotheses require **both** null hypotheses to be rejected for the trial intervention to be a "success".	◯	◯	◯
The trial hypotheses require **either** null hypothesis to be rejected for the trial intervention to be a "success".	◯	◯	◯

Consider a parallel group trial (two treatment arms) where the primary outcome is measured at five time points. The primary endpoint is the overall effect over the entire time period. Would you implement a multiple testing procedure for the primary outcome?

◯ Yes ◯ No ◯ Unsure

Consider a parallel group trial (two treatment arms) with multiple secondary outcomes. Would you implement a multiple testing procedure for the secondary outcomes?

◯ Yes ◯ No ◯ Unsure

Would the type of outcomes (efficacy, safety, cost-effectiveness) have an impact on your response to the above question?

◯ Yes ◯ No ◯ Unsure

Please give any rationale/further details relevant to this section:

Multiple testing in RCTs

APPROACHES TO ADDRESS MULTIPLICITY: MULTIPLE TREATMENT COMPARISONS

This section focuses on pragmatic RCTs (i.e. "larger" trials where there is an expectation that results will be generalisable to the wider NHS).

Would you consider implementing a multiple testing procedure to address multiplicity arising from making multiple treatment comparisons (e.g. >2 treatment arms in a parallel group trial)?

◯ Yes ◯ No ◯ Unsure

If **YES**, complete the rest of this section. If **NO**, go to next section.

Consider a parallel group trial with three treatment arms, where all comparisons are of interest. Would you implement a multiple testing procedure for the primary outcome in the following scenarios?

	Yes	No	Unsure
Two of the treatment arms are related, *e.g.: Group 1=placebo, Group 2=low dose of antibiotics Group 3=high dose of antibiotics*	◯	◯	◯
The three treatment arms are unrelated, including one placebo arm, *e.g. Group 1=placebo Group 2=pre-surgery antibiotics Group 3=pre-surgery exercise program*	◯	◯	◯
The three treatment arms are unrelated, but all are active treatments, *e.g. Group 1=pre-surgery education group Group 2=pre-surgery antibiotics Group 3=pre-surgery exercise program*	◯	◯	◯

Would you be more likely to implement a multiple testing procedure if the number of treatment arms was increased (i.e. >3)?

◯ Yes ◯ No ◯ Unsure

Please give any rationale/further details relevant to this section:

APPROACHES TO ADDRESS MULTIPLICITY: SUBGROUP ANALYSES

This section focuses on pragmatic RCTs (i.e. "larger" trials where there is an expectation that results will be generalisable to the wider NHS).

Would you consider implementing a multiple testing procedure to address multiplicity arising from performing multiple subgroup analyses?

◯ Yes ◯ No ◯ Unsure

If **YES**, complete the rest of this section. If **NO**, go to next section.

Consider a parallel group trial (two treatment arms) with multiple subgroup analyses performed for the primary outcome. Would you implement a multiple testing procedure for these subgroup analyses in the following scenarios?

	Yes	No	Unsure
The subgroup analyses were pre-specified in the study protocol	◯	◯	◯
The subgroup analyses were detemined post-hoc	◯	◯	◯
The subgroup analyses were specified for the following reasons:<br>a) to confirm biological plausibility,<br>b) to confirm existing hypotheses, AND<br>c) to show subgroup effects for supporting decision making in target populations.	◯	◯	◯

Would you be more likely to implement a multiple testing procedure if the number of subgroup analyses was increased (e.g. ten subgroup analyses vs two)?

◯ Yes ◯ No ◯ Unsure

Please give any rationale/further details relevant to this section:

[ ]

APPROACHES TO ADDRESS MULTIPLICITY: INTERIM ANALYSES

This section focuses on pragmatic RCTs (i.e. "larger" trials where there is an expectation that results will be generalisable to the wider NHS).

Would you implement a multiple testing procedure if interim analysis(es) were pre-specified in the study protocol?

◯ Always ◯ Sometimes ◯ Never ◯ Unsure

What factors influence your decision making around when/how to implement multiple testing procedures for interim analyses?

[ ]

Which multiple testing procedures have you used for interim analyses?

[ ]

APPROACHES TO ADDRESS MULTIPLICITY: OTHER TRIAL DESIGN CONSIDERATIONS

This section focuses on pragmatic RCTs (i.e. "larger" trials where there is an expectation that results will be generalisable to the wider NHS).

Indicate whether the following trial design features would affect your decision to implement a multiple testing procedure:

	Yes	No	Unsure
Trial design (e.g. cluster, factorial, crossover)	○	○	○
Hypothesis type (e.g. superiority, non-inferiority, equivalence)	○	○	○
Intervention type (e.g. complex, behavioural, pharmacological)	○	○	○
Imbalanced trial allocation (e.g. 2:1)	○	○	○

Please give further details to your responses above, or any other trial design features that would affect your approach to multiplicity:

[ ]

STATISTICAL METHODS TO ADDRESS MULTIPLICITY

This section focuses on pragmatic RCTs (i.e. "larger" trials where there is an expectation that results will be generalisable to the wider NHS).

Indicate whether you have used the following multiple testing procedures:

	Often used	Occasionally used	Never used
Bonferroni procedure	○	○	○
Simes procedure	○	○	○
Holm step-down procedure	○	○	○
Hochberg step-up procedure	○	○	○
Hommel procedure	○	○	○
Dunnett procedure	○	○	○
Fixed-sequence procedure	○	○	○
Fallback procedure	○	○	○
Serial gatekeeping procedure	○	○	○
Parallel gatekeeping procedure	○	○	○
Other gatekeeping procedure	○	○	○
Graphical methods (e.g. recycling significance levels)	○	○	○

Other (please specify)

[ ]

Please give further details (if possible) for choices of methods/situations they have been used in:

[ ]

OTHER INFORMATION

What do you think are the common problem areas for multiplicity? Where is research needed?

Would you be happy to be contacted to discuss your responses to this survey further? This could be in email/teleconference form (whichever is preferable to you), and there may be opportunities for co-authorship on the intended publication summarising the results of this survey. If so please give your contact details below:

Any other comments/information:

You have reached the end of the survey (other than an optional section that follows).
Thank you very much for your contribution

OPTIONAL SECTION: TYPICAL RESEARCH QUESTIONS

This section focuses on typical research questions, and therefore situations in which multiplicity arises.

Of the RCTs currently being managed by your unit (including RCTs in set-up, recruitment and analysis stages), give the **approximate number and/or percentage** that are pragmatic (i.e. "larger" trials where there is an expectation that results will be generalisable to the wider NHS):

Of the pragmatic trials managed by your unit, give the**approximate number and/or percentage** that have:

Multiple primary outcomes

Multiple secondary outcomes

Repeated measurements on the same patient for the primary outcome

More than two treatment groups (parallel group trials)

Planned subgroup analyses

Planned interim analyses

Thank you again for your contribution
