# Supplementary material for: Approaches to multiplicity in publicly funded pragmatic randomised controlled trials: a survey of clinical trials units and a rapid review of published trials
Source: BMC Med Res Methodol. 2022 Feb 6;22:39. doi: 10.1186/s12874-022-01525-9 (PMC8818238; doi:10.1186/s12874-022-01525-9)
Supplement: Supplementary file 3 — Additional file 3: Table S1. Review: characteristics of trials that did/did not have multiple primary outcomes. Table S2. Review: characteristics of trials that did/did not perform multiple treatment comparisons. Table S3. Review: characteristics of trials that did/did not perform subgroup analyses. Table S4. Review: characteristics of trials that did/did not perform interim analyses. Table S5. Survey: existing practices in CTUs to address multiplicity. Table S6. Survey: effect of other trial design features on the approach to multiplicity. Table S7. Survey: statistical methods used to address multiplicity. [file 12874_2022_1525_MOESM3_ESM.pdf]

Additional file 3

Supplementary tables and figures

Table S1 Review: characteristics of trials that did/did not have multiple primary outcomes

Characteristic		Multiple primary outcomes (n=28)		Single primary outcome (n=110)	
Journal	Annals Int Med	1/28	4%	3/110	3%
	BMJ	4/28	14%	7/110	6%
	JAMA	7/28	25%	21/110	19%
	Lancet	6/28	21%	24/110	22%
	NEJM	5/28	18%	31/110	28%
	NIHR HTA	4/28	14%	13/110	12%
	PlosMED	1/28	4%	11/110	10%
Trial design	Parallel group: 2 treatment groups	17/28	61%	77/110	70%
	Parallel group: >2 treatment groups	6/28	21%	12/110	11%
	Cluster randomised	3/28	11%	20/110	18%
	Crossover	1/28	4%	1/110	1%
	Factorial	1/28	4%	4/110	4%
	Stepped wedge	0/28	0%	2/110	2%
	Non-inferiority	6/28	21%	12/110	11%
	Equivalence	0/28	0%	1/110	1%
Total number of randomised participants, median (IQR)		485	(291, 1899)	606	(319, 2157)

Table S2 Review: characteristics of trials that did/did not perform multiple treatment comparisons

Characteristic		More than two treatment groups (n=23)		Two treatment groups (n=115)	
Journal	Annals Int Med	1/23	4%	3/115	3%
	BMJ	1/23	4%	10/115	9%
	JAMA	4/23	17%	24/115	21%

	Lancet	7/23	30%	23/115	20%
	NEJM	5/23	22%	31/115	27%
	NIHR HTA	3/23	13%	14/115	12%
	PlosMED	2/23	9%	10/115	9%
Trial design	Parallel group: 2 treatment groups	0/23	0%	94/115	82%
	Parallel group: >2 treatment groups	18/23	78%	0/115	0%
	Cluster randomised	3/23	13%	20/115	17%
	Crossover	0/23	0%	2/115	2%
	Factorial	5/23	22%	0/115	0%
	Stepped wedge	0/23	0%	2/115	2%
	Non-inferiority	2/23	9%	16/115	14%
	Equivalence	0/23	0%	1/115	1%
Total number of randomised participants		722	(374, 5177)	513	(300, 2043)

Table S3 Review: characteristics of trials that did/did not perform subgroup analyses

		Subgroup analyses		No subgroup analyses	
**Characteristic**		**performed (n=85)**		**performed (n=53)**	
Journal	Annals Int Med	2/85	2%	2/53	4%
	BMJ	8/85	9%	3/53	6%
	JAMA	19/85	22%	9/53	17%
	Lancet	16/85	19%	14/53	26%
	NEJM	19/85	22%	17/53	32%
	NIHR HTA	14/85	16%	3/53	6%
	PlosMED	7/85	8%	5/53	9%
Trial design	Parallel group: 2 treatment groups	61/85	72%	33/53	62%
	Parallel group: >2 treatment groups	9/85	11%	9/53	17%
	Cluster randomised	13/85	15%	10/53	19%
	Crossover	1/85	1%	1/53	2%
	Factorial	2/85	2%	3/53	6%
	Stepped wedge	2/85	2%	0/53	0%

	Non-inferiority	7/85	8%	11/53	21%
	Equivalence	1/85	1%	0/53	0%
Total number of randomised participants		631	(350, 2555)	470	(290, 1241)

Table S4 Review: characteristics of trials that did/did not perform interim analyses

Characteristic		Interim analyses performed (n=41)		No interim analyses performed (n=97)	
Journal	Annals Int Med	0/41	0%	4/97	4%
	BMJ	0/41	0%	11/97	11%
	JAMA	7/41	17%	21/97	22%
	Lancet	11/41	27%	19/97	20%
	NEJM	18/41	44%	18/97	19%
	NIHR HTA	5/41	12%	12/97	12%
	PlosMED	0/41	0%	12/97	12%
Trial design	Parallel group: 2 treatment groups	33/41	80%	61/97	63%
	Parallel group: >2 treatment groups	3/41	7%	15/97	15%
	Cluster randomised	3/41	7%	20/97	21%
	Crossover	0/41	0%	2/97	2%
	Factorial	2/41	5%	3/97	3%
	Stepped wedge	1/41	2%	1/97	1%
	Non-inferiority	4/41	10%	14/97	14%
	Equivalence	0/41	0%	1/97	1%
Total number of randomised participants		782	(363, 3096)	500	(306, 1638)

Table S5 Survey: existing practices in CTUs to address multiplicity

Characteristic		n/N	%
At what stage is the approach to address multiplicity in RCTs usually determined?	Design (stated in application)	15/27	56%
	Design (stated in protocol)	2/27	7%
	Design (stated in SAP)	6/27	22%
	Analysis	1/27	4%
	Varies	3/27	11%
	Never determined	0/27	0%
If determined at the design stage, have there been circumstances when post-hoc decisions have been made about multiplicity?[1]	Yes	10/26	38%
	No	12/26	46%
	Unsure	4/26	15%
Is the approach to multiplicity in your CTU?[2]	Standard across all trials	5/27	19%
	Bespoke approach that varies from trial to trial	21/27	78%
	Other	1/27	4%
Would your approach to multiplicity vary according to how pragmatic the trial is?[3]	Yes	10/27	37%
	Possibly	9/27	33%
	No	4/27	15%
	Unsure	4/27	15%

Notes:

[1] For those that responded "yes", further comments have been categorized into: oversight committee's request (n=2), post-hoc outcomes or analyses added (n=3), peer-review suggestions (n=2), changes made when writing SAP (n=2), miscellaneous (n=1)

[2] Further comments have been categorized into:

- *Standard across all trials: standard approach for translational studies only (n=1)*
- *Bespoke approach: context specific (n=7), differs for multi-arm trials (n=2), varies between statisticians (n=1), different approach for trials seeking licensing approval (n=1)*
- *Other: by therapeutic area (n=1)*

[3] Further comments have been categorized into:

- *Responded yes/possibly: adjustments less necessary for early phase trials (n=5), adjustments less necessary for pragmatic trials (n=2), miscellaneous (n=3)*
- *Responded no: based on design, not phase (n=1), miscellaneous (n=1)*
- *Responded unsure: adjustments less necessary for pragmatic trials (n=1), mainly do pragmatic trials (n=2)*

Table S6 Survey: effect of other trial design features on the approach to multiplicity

Situation	Yes		No		Unsure	
	n/N	%	n/N	%	n/N	%
Would the following trial design features affect your decision to implement a MTP?						
Trial design (e.g. cluster, factorial, crossover)	9/26	35%	14/26	54%	3/26	12%
Hypothesis type (e.g. superiority, non-inferiority, equivalence)	5/26	19%	17/26	65%	4/26	15%
Intervention type (e.g. complex, behavioural, pharmacological)	6/26	23%	18/26	69%	2/26	8%
Imbalanced trial allocation (e.g. 2:1)	1/26	4%	21/26	81%	4/26	15%

Notes:

Other information given was classified as follow: factorial design may affect decision to adjust (n=3), non-inferiority/equivalence design requires thought (n=1), none of these factors would affect decision to adjust (n=7)

Table S7 Survey: statistical methods used to address multiplicity

Method	Often used		Occasionally used		Never used	
	n/N	%	n/N	%	n/N	%
Bonferroni procedure[1]	8/27	30%	16/27	59%	3/27	11%
Simes procedure	0/27	0%	4/25	16%	21/25	84%
Holm step-down procedure[2]	0/27	0%	8/25	32%	17/25	68%
Hochberg step-up procedure[2]	0/27	0%	6/25	24%	19/25	76%
Hommel procedure	0/27	0%	2/25	8%	23/25	92%
Dunnett procedure[3]	1/26	4%	13/26	50%	12/26	46%
Fixed-sequence procedure	1/26	4%	5/26	19%	20/26	77%

Fallback procedure	1/25	4%	1/25	4%	23/25	92%
Serial gatekeeping procedure[2]	1/24	4%	5/24	21%	18/24	75%
Parallel gatekeeping procedure[2]	1/23	4%	1/23	4%	21/23	91%
Other gatekeeping procedure[2]	1/24	4%	3/24	13%	20/24	83%
Graphical methods (e.g. recycling significance levels)	0/27	0%	3/24	13%	21/24	88%

Notes:

Six other methods were mentioned: Peto-heybittle, group sequential methods, alpha spending, O'Brien & Fleming, Pocock, Posch combination test.

Comments given regarding the situation methods were used in (for details see appendix):

[1] Used in a range of scenarios, generally multiple primary outcomes and/or treatment comparisons (n=4 comments)

[2] Used for multiple outcomes which can be ordered hierarchically (n=1 comment)

[3] Used for multiple treatment comparisons (n=3 comments)

Peto-heybittle used for interim analysis (n=1 comment)

Choice of method depends on the trial design and reason for adjusting (n=2 comments)
